# Intra-fraction setup variability: IR optical localization vs. X-ray imaging in a hypofractionated patient population

**DOI:** 10.1186/1748-717X-6-38

**Published:** 2011-04-15

**Authors:** Maria Francesca Spadea, Barbara Tagaste, Marco Riboldi, Eleonora Preve, Daniela Alterio, Gaia Piperno, Cristina Garibaldi, Roberto Orecchia, Antonio Pedotti, Guido Baroni

**Affiliations:** 1 Department of Experimental and Clinical Medicine, Università degli Studi Magna Græcia, Catanzaro, Italy; 2 Department of Bioengineering, Politecnico di Milano University, Milano, Italy; 3 Centro Nazionale di Adroterapia Oncologica, Pavia, Italy; 4 Medical Physics Department, Istituto Europeo di Oncologia, Milano, Italy; 5 Radiotherapy Division, Istituto Europeo di Oncologia, Milano, Italy

## Abstract

**Background:**

The purpose of this study is to investigate intra-fraction setup variability in hypo-fractionated cranial and body radiotherapy; this is achieved by means of integrated infrared optical localization and stereoscopic kV X-ray imaging.

**Method and Materials:**

We analyzed data coming from 87 patients treated with hypo-fractionated radiotherapy at cranial and extra-cranial sites. Patient setup was realized through the ExacTrac X-ray 6D system (BrainLAB, Germany), consisting of 2 infrared TV cameras for external fiducial localization and X-ray imaging in double projection for image registration. Before irradiation, patients were pre-aligned relying on optical marker localization. Patient position was refined through the automatic matching of X-ray images to digitally reconstructed radiographs, providing 6 corrective parameters that were automatically applied using a robotic couch. Infrared patient localization and X-ray imaging were performed at the end of treatment, thus providing independent measures of intra-fraction motion.

**Results:**

According to optical measurements, the size of intra-fraction motion was (*median ± quartile*) 0.3 ± 0.3 mm, 0.6 ± 0.6 mm, 0.7 ± 0.6 mm for cranial, abdominal and lung patients, respectively. X-ray image registration estimated larger intra-fraction motion, equal to 0.9 ± 0.8 mm, 1.3 ± 1.2 mm, 1.8 ± 2.2 mm, correspondingly.

**Conclusion:**

Optical tracking highlighted negligible intra-fraction motion at both cranial and extra-cranial sites. The larger motion detected by X-ray image registration showed significant inter-patient variability, in contrast to infrared optical tracking measurement. Infrared localization is put forward as the optimal strategy to monitor intra-fraction motion, featuring robustness, flexibility and less invasivity with respect to X-ray based techniques.

## 1. Background

Over the last few years, the development of Image Guided Radiation Therapy (IGRT) technologies has resulted in the design and realization of systems allowing precise patient setup and monitoring at each therapy fraction [1-3]. The rationale is related to dose escalation and hypo-fractionated protocols, which require the precise localization of the target throughout the treatment. Morphological changes, tumor shrinkage and organ motion effects lead to inter-fraction variations that potentially jeopardize the dose delivered to the target volume, as defined on the treatment planning CT. Recently, different in room imaging modalities (stereoscopic X-rays, Kilo-Voltage and Mega-Voltage cone-beam CT, megavoltage CT, CT on rail, ultrasonography) have been made available for the implementation of IGRT protocols relying on bony anatomy and/or soft tissue contrast [4-9]. The availability of these technologies provides the minimization of patient setup errors and the capabilities to evaluate the need for re-planning, in the framework of and Adaptive Radiotherapy (ART) approach [10]. Along with inter-fraction variations, intra-fraction uncertainties due to physiological (respiration, swallowing, heartbeat and peristalsis) and/or random movements of the patient may also influence the treatment quality, especially for extra-cranial sites. This requires the definition of specific procedures for the verification of intra-fractional patient motion as part of IGRT treatment protocols.

When imaging techniques are used, the assessment of intra-fraction uncertainties in most cases is measured off-line at the end of irradiation. Actual real-time patient monitoring is usually achieved by tracking external surrogates, like Infra-Red (IR) markers [11,12] or the entire skin surface [13,14] or by acquiring the position of implanted seeds. These latter can either be radio-opaque markers, to be detected by fluoroscopy, or electromagnetic transponders, which can be localized continuously with non ionizing radiation [15-18]. The main drawbacks of implanted fiducials are related to the fact that the procedure is invasive and may imply non-negligible risks for the patient [19,20]. Moreover, inter-fraction seed migration can compromise the accuracy of using implanted fiducials as surrogates [21]. On the other hand, IR markers or surface detection represent non invasive techniques but they provide information related to distant surrogates from the target. For this reason, their application needs to be supported by studies aiming at understanding their reliability with respect to image-based procedures.

In 2006 Linhout *et al. *[22] investigated the capabilities of the ExacTrac X-ray 6D system (BrainLab, Germany) in detecting intra-fraction motion in 13 head and neck patients treated with IMRT. The system from BrainLab consists of 2 infrared (IR) TV cameras for the 3-D localization of 5-7 surface markers, and stereoscopic X-ray imaging for the automatic matching of daily images and digitally reconstructed radiographs (DRR). The authors found significant discrepancies between the corrective parameters suggested by the two sub-systems for intra-fraction measurement. Their conclusion was that in the cranial district, where a large percentage of bony structures is clearly visible, X-ray registration is more accurate and reliable to detect intra-fraction movements of the head within the immobilization mask.

In this work, we extend the analysis to frame-based and frameless hypo-fractionated (1-to-4 sessions) radiation therapy including cranial and extra-cranial treatment sites. An off-line analysis was performed on the log files storing the position of markers before and after treatment to measure 3D displacements. Stereoscopic X-ray images were acquired and matched before and after treatment to measure bony anatomy shifts. The specific aim of our study was the multimodal measurement of intra-fraction variations and the exploration of optimal strategies for monitoring the intra-fraction setup variability in high precision radiation therapy.

## 2. Materials and methods

### Patients selection

We randomly selected 87 patients treated between May 2007 and March 2009 with hypo-fractionated stereotactic radiotherapy. The number of analyzed therapy sessions was 151 out the total of 231. Time limitations in the clinical routine and the absence of dedicated personnel on a regular basis did not allow us to acquire data at every fraction. Details about the patient population are presented in Table 1.

**Table 1:** Patient population

	Number of patients	Number of treatment fractions	Number of analyzed fractions	Dose per fraction (min-max) [Gy]
**Cranial**	18	33	31	15-21

**Abdomen**	26	77	52	8-15

**Lung**	43	121	68	8-18

### Target definition and irradiation technique

The treatment plan was calculated on a planning CT image set acquired with 3 mm slice thickness, using the BrainScan software (BrainLab, Germany). In cranial patients, isotropic margins ranging between 3 mm and 5 mm were added to the CTV (Clinical Target Volume) to define the PTV (Planned target volume). For extra-cranial treatments, anisotropic margins were defined on the basis of a breath hold CT scan acquisition around the target region, thus taking into account the tumor excursion from exhale to inhale (Internal Margin). A slow CT scan was also acquired to ensure that tumor motion, during normal breathing, was included in the PTV. Additional 3 mm were added, in order to take into account setup uncertainties. The dose was normalized at the ICRU (International Commission on Radiation Units and Measurements) reference point in order to obtain that the 95% of PTV was covered by the 95% isodose. The treatment was delivered with the support of a 3 mm multileaf collimator from Brainlab.

### Patient setup

**Figure 1:**
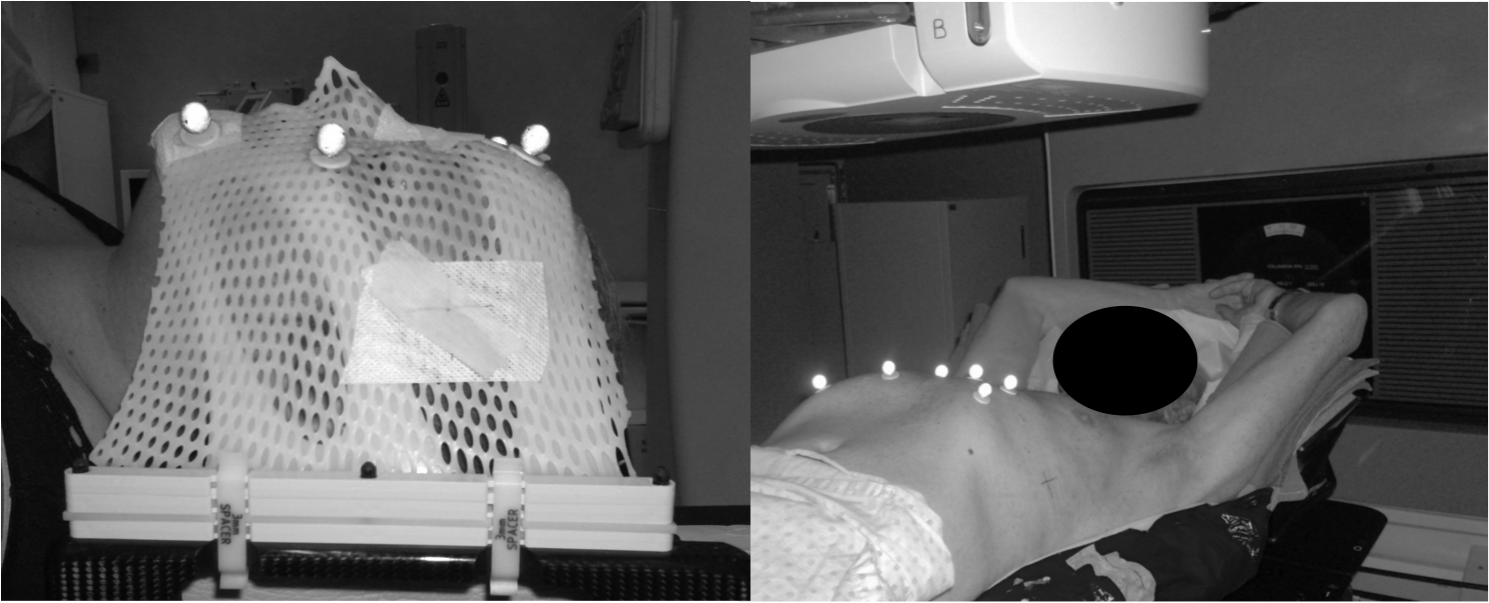
**Patient set up and immobilization**. Panel A, patient setup for cranial treatment. The thermoplastic mask is fitted with 7 IR markers for stereotactic localization. Panel B, patient setup for body treatments. A vacuum cushion is modeled on the subject who lies aided by an arm support. For body treatments a leg support device is also used for immobilization purposes. In both cases, markers are placed on patient's skin with a biocompatible tape.

The clinical protocol was designed and approved to monitor intra-fraction setup variability in selected patients. Head and neck patients (see Figure 1, left panel) were immobilized with a personal thermoplastic mask (the Head and Neck Frameless SRS from BrainLab) fitted with 6-7 IR markers for stereotactic localization. For extra-cranial treatments (see Figure 1, right panel), a vacuum cushion (Vac-Lok Cushions from CIVCO) was modeled on the body and arm/leg supports were used for lung/abdomen patients, respectively. Markers were placed on the patient skin without the use of any stereotactic frame, as described by Baroni *et al. *[12].

Patient setup was driven by the ExacTrac X-Ray system, an IGRT device featuring two sub-components; 1) an Infra-Red (IR) optoelectronic localizer and 2) a radiographic kV X-ray imaging device in double oblique projection. The IR localization features real time detection (30 Hz) of passive spherical markers (10 mm of diameter) with a ± 0.3 mm localization error. The field of view of kV images is 20.4 × 20.4 cm^2^, sampled in 512 × 512 pixel units. In our protocol, image registration is performed on the basis of bony anatomy matching (skull or spine). The user can manually exclude up to 70% of the image in order to remove ambiguous structures (like ribs, external marker projections, organs shadows etc.) from the registration process. The outcomes of image fusion are 6 corrective parameters that are applied through the robotic couch (ExacTrac Remote couch by Brainlab). A comprehensive technical description of the system can be found in Jin *et al. *[23].

At each therapy fraction, automatic patient alignment was perfomed by the optical system along the three linear directions (Left-Right, LR, Cranio-Caudal, CC, Antero-Posterior, AP). After that, two orthogonal kV images were acquired and automatically matched to DRR for computing setup corrections in 6 degrees of freedom (Dof, 3 translations and 3 rotations) relying on bony anatomy. The correction was then performed through the 6 Dof robotic couch. A second X-ray acquisition was performed to measure the residual errors according to the imaging system. If residual translations and rotations were found below 1 mm and 1° respectively, the patient position was considered acceptable for treatment; otherwise the procedure was repeated iteratively to improve patient setup.

### Intra-fraction variation monitoring and data analysis

Following patient setup procedures and before irradiation started, the 3D location of external markers (*PreIR*) was acquired and averaged over at least 2 breathing cycles (8-10 seconds). The *PreIR *configuration represents the reference position for monitoring intra-fraction variations in our analysis, including the position of the target, which was automatically estimated by the ExacTrac software from the current arrangement of markers.

**Figure 2:**
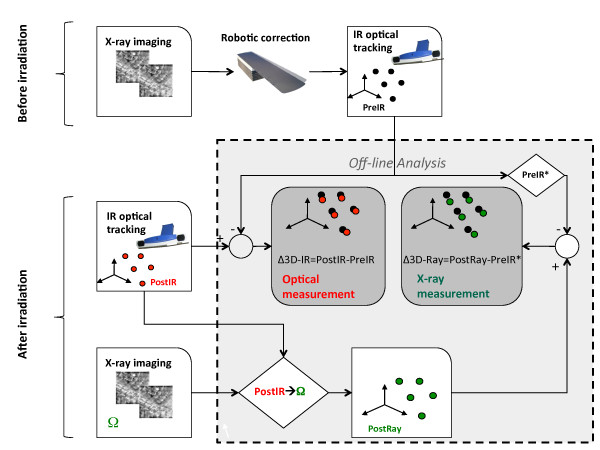
**Workflow of data acquisition and analysis**. The 3D position of external surrogates was acquired before and after the irradiation. Patient was also imaged trough X-ray imaging before and after the treatment. Data were analyzed off line to measure the intra-fraction motion according to the two subsystem.

In Figure 2, the workflow for the assessment of intra-fraction motion is depicted. The time interval between start and end of treatment ranged between 5 and 10 minutes. As soon as irradiation ended, IR markers were again localized and stored, for the definition of the post-irradiation configuration (*PostIR*), that was averaged over the same time duration (8-10 seconds) that was used for *PreIR*. A post irradiation set of X-ray images was also acquired and registered to DRRs, for the estimation of post-irradiation 6 Dof roto-translation parameters (Ω) describing image-based intra-fraction motion. Off-line analysis of intra-fraction motion was expressed in terms of positional variations between pre and post irradiation and was performed following two approaches:

1. Optical measurement: 3D displacements between *PreIR *and *PostIR*.

2. X-ray measurement: for consistency sake intra-fraction motion was quantified in terms of displacements of surface control points, accounting for information provided by pre-irradiation and post-irradiation image registration. This was achieved as follows:

• Roto-translation of the *PreIR *configuration, according to the residual corrective parameters provided by image matching before irradiation; this resulted in the PreIR* configuration of control points, accounting for residual patient setup errors as detected by X-ray imaging

• Roto-translation of the *PostIR *configuration according to post-irradiation image registration (**Ω **correction vector), leading to *PostXRay *configuration.

• Calculation of 3D displacements between PreIR* and *PostXRay*.

A further analysis was performed on the target location. The center of mass of the tumor was estimated by applying the weighted strategy algorithm proposed by Riboldi *et al.*[24]. The Euclidean distance between post-irradiation and reference target positions was calculated for both *PostIR *and *PostXray *configuration.

## 3. Results

**Figure 3:**
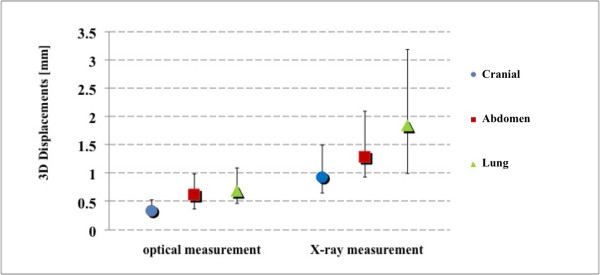
**Intra-fraction error on external markers**. 3D mismatches on control points before and after the irradiation according to the two different measurement approaches.

The normality test rejected the hypothesis of normal distribution in the population of 3D fiducial displacements. For this reason, data were analyzed following a non-parametric statistical approach. Due to statistically significant differences (Kruskal-Wallis test followed by post hoc Siegel-Tukey test [25], p < 10^-6^) results for cranial, abdomen and lung patients are reported separately. In Figure 3, results relative to the IR-based and X-ray-based intra-fraction motion measurements are reported. Pre-versus post-irradiation 3D displacements of external fiducials (median ± quartile - 95^th ^percentile) were 0.3 ± 0.3 mm - 1.0 mm, 0.6 ± 0.6 mm - 2.1 mm, 0.7 ± 0.6 mm - 1.4 mm (cranial, abdomen and lung patients respectively) for optical measurements. Conversely, X-ray detected values measured 0.9 ± 0.8 mm - 2.9 mm, 1.3 ± 1.2 mm - 3.9 mm, 1.8 ± 2.2 mm - 7.1 mm.

The Wilcoxon matched pair test demonstrated statistical difference between optical and X-ray systems in each patient population (p < 10^-6^). As reported in Table 2 the most relevant difference between optical and X-ray measurements was found in the Left-Right direction for cranial patients, and in the Cranio-Caudal direction for extra-cranial patients.

**Table 2:** Mean and standard deviation [mm] errors along left-right (LR), cranio-caudal (CC) and antero-posterion (AP) direction resulted after optical and X-ray measurement.

	Optical			X-Ray		
	**LR**	**CC**	**AP**	**LR**	**CC**	**AP**

**Cranial**	0.07 (0.40)	0.06 (0.28)	-0.11 (0.10)	0.00 (1.25)	-0.01 (0.45)	0.00 (0.49)

**Abdomen**	-0.10 (0.46)	-0.13 (0.59)	-0.14 (0.67)	-0.25 (1.40)	-0.23 (1.06)	0.49 (1.13)

**Lung**	0.01 (0.50)	0.00 (0.63)	-0.15 (0.57)	0.25 (1.73)	-0.18 (2.24)	-0.04 (1.61)

**Figure 4:**
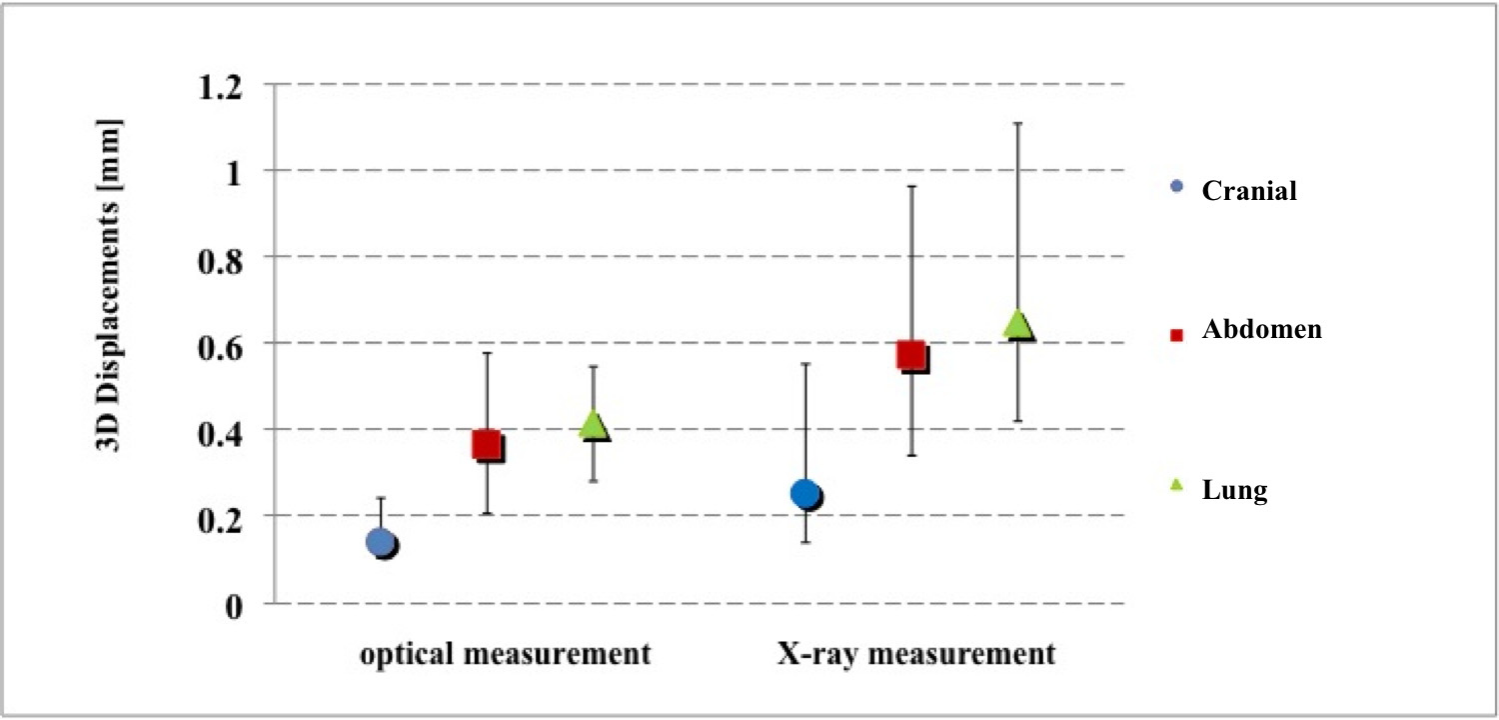
**Estimation of intra-fraction error on target**. 3D estimated intra-fraction motion of the target according to the two different measurement approaches.

Figure 4 shows the Euclidean distance between the estimated position of the target before and after irradiation. Median ± quartile - 95th percentile values were 0.1 ± 0.1 mm - 0.5 mm, 0.4 ± 0.4 mm - 1.1 mm, 0.4 ± 0.3 mm - 1.3 mm for optical measurements, vs. 0.3 ± 0.4 mm - 1.2 mm, 0.6 ± 0.6 mm - 1.6 mm, 0.7 ± 0.7 mm - 2.5 mm, for X-ray measurements, in cranial, abdomen and lung patients respectively. Also in this case a statistical difference was found between the two monitoring systems (p < 10^-3^).

**Figure 5:**
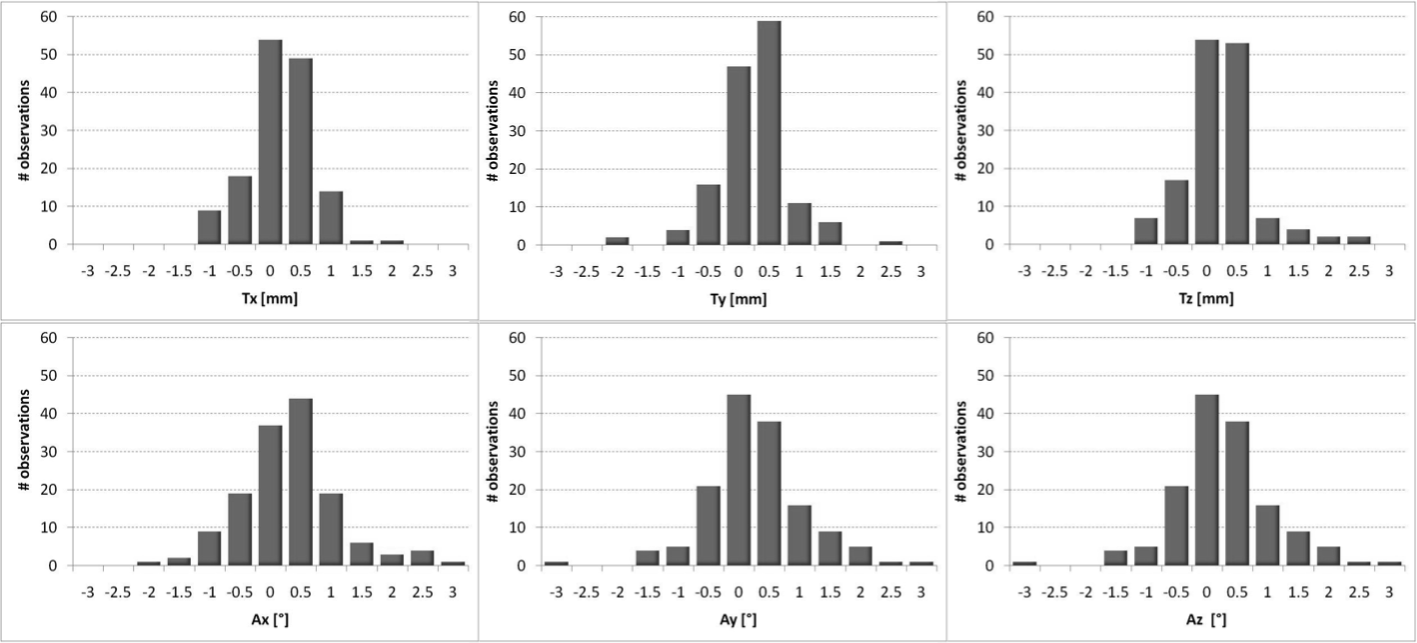
**6 dof corrective parameters**. Frequency distribution plots of the linear (Tx, Ty, Tz) and angular deviations (Ax, Ay, Az) resulting from kV X-ray images and DRR matching after irradiation. Bars are centered on labels and ranges over a 0.5 mm interval.

Figure 5 reports the frequency-histograms of the 6 verification parameters (Ω) for all patients, as detected by image registration after treatment. In 58 out of 151 analyzed fractions, one or more parameters were larger than the threshold of clinical acceptability established in our clinical protocol (1 mm and 1° for linear and angular deviations respectively).

One outlier, which is not displayed in the plots, showed 12.8 mm translation along the left-right direction, with acceptable values for the other directions (up to 2.2 mm translation in AP-direction and up to 0.6° yaw rotation). The optical system did not detect relevant shifts for this case.

## Discussion

In this work, we measured intra-fraction motion in hypo-fractionated radiotherapy using a multimodal approach. Our main goal was to assess the quality of patient immobilization during treatment and to highlight the optimal measurement strategy (IR localization vs. X-ray imaging). It is important to underline three relevant aspects of the implemented methodology:

1. since extra-cranial treatments are performed in free breathing conditions, IR data were collected and averaged over at least two respiratory cycles to compensate possible respiration motion effects in a short time window. The effect of respiration movements was furthermore evaluated by measuring the standard deviation (std) of marker positions over each acquisition. The mean standard deviation ranged between 0.3 and 0.5 mm in the extra-cranial patient population. These values are due to the fact that most of the IR markers (4-5 over 7) were placed in correspondence of stable landmarks, like upper thorax or pelvis, thus leading to a robust measurement of patient position.

2. The cranial patient population potentially represents an ideal situation, as intra-fraction motion is less relevant. However, the presence of the thermoplastic mask may represent a limitation because markers are typically placed onto the mask in our protocol. Therefore, the discrepancies found between the two measurements approaches can be due in part to movements of the patient within the mask, as suggested by Linthout *et al. *[22].

3. The X-ray measurements were depurated from setup residuals, computed before treatment by means of image registration. This gave us more robustness in understanding and analyzing an X-ray based quantitative measurement of intra-fraction variations.

The analysis was performed off-line, by analyzing both the log files of markers position and the X-ray images stored immediately before and after irradiation. Compared to the methodology proposed by Linthout *et al*., the main differences in our data analysis were the following:

1. In the work by Linthout *et al*. the intra-fraction motion monitored by the optical localizer was evaluated in terms of the 6 Dof corrective parameters estimated by the Brainlab software. Here, we assessed the residual displacements on each external marker after optical measurements and then we estimated the isocenter position from the configuration of fiducials. This allowed us also to explore potential deformations in the configuration of markers, in order to test its reliability in patient setup control.

2. In Linthout *et al*. the comparison between the two sub-systems was performed by evaluating the corrective parameters coming from external point registration and image fusion. This kind of analysis has a conceptual flaw since an indeterminate number of roto-translations are able to match 2 different configurations in space at the same uncertainty level. Here, we roto-translated the external configuration of marker points according to image fusion and then we compared the 2 fiducial sets, point by point, to precisely examine the difference between the 2 approaches.

Measurements performed by the optical localizer showed on average sub-millimetric intra-fraction motion for both extra-cranial and cranial treatments. These results were confirmed when looking at target position, as estimated according to the external marker configuration under a rigid body assumption. Target position resulted essentially stable, with average intra-fraction motion within 1 mm. On the basis of these results, we can assume that immobilization devices and the automation of setup procedures help the patient to be comfortable and stable, thus leading to small intra-fraction variations.

**Figure 6:**
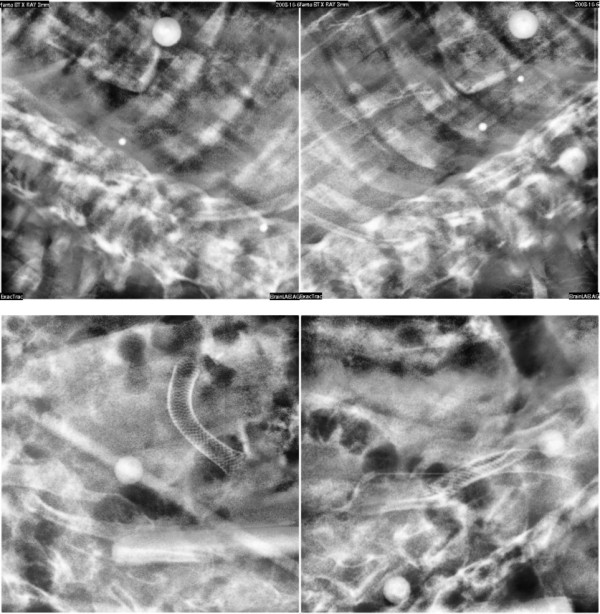
**x-ray image quality**. *Upper panels*: X-ray images acquired on an anthropomorphic radio-equivalent phantom. *Lower panels*: X-ray images acquired on a patient after treatment.

When comparing optical versus X-ray measurements, differences were on average 1-1.5 mm, with worst results in lung cases. It should be noted from Figures 3 and 4 that X-ray imaging resulted in larger intra-fraction motion compared to IR localization, with increased inter-patient variability. Such discrepancies should be judged against the intrinsic accuracy of the two systems (around 0.3 mm for optical localization [23] and half CT slice thickness for image matching, 1.5 mm in our case). Digital image noise and image artifacts might occasionally originate considerable errors in registration as testified by the outlier case that we reported in the results section (12.8 mm linear shift). The influence of image quality on the reliability of image registration was also demonstrated during internal commissioning studies on an anthropomorphic radio-equivalent phantom. In Figure 6, we report a comparison between images acquired on phantom and patients. Phantom studies showed no appreciable difference between the optical localizer and X-ray image registration in 10 repeated measurements. In the patient case, the image is clearly more blurred and noisy and image registration led to a discrepancy of about 2 mm in target localization compared to optical measurements. Our conclusion is that the quality of X-ray images must be accurately verified when using image registration for intra-session monitoring, as the sensitivity is extremely case specific.

## Conclusions

Patient setup verification should rely on multimodal monitoring systems (X-ray and IR optical) for the highest reliability in detecting and correcting geometric uncertainties. The reported analysis shows that optical tracking is able to provide robust measurement for the real-time detection of intra-fraction variations.

## List of abbreviations

AP: Antero-Posterior; ART: Adaptive Radiation Therapy; CBCT: Cone Beam Computed Tomography; CC: Cranio-Caudal; CT Computed Tomography; Dof; degrees of freedom; IGRT: Image Guided Radiation Therapy; IMRT: Intensity Modulated Radiation Therapy; IR: Infra-Red; kV: kilo Voltage; LR: Left-Right; MV: Mega Voltage; PostIR: 3D Marker position detected by the IR localizer after treatment; PostXRay: PostIR roto-translated according to the corrective parameters (Ω) estimated by image registration after treatment; PreIR: 3D Marker position detected by the IR localizer before treatment; PreIR*: PreIR roto-translated according to the verification parameters estimated by image registration before treatment

## Authors' contributions

MFS had primary role in study design, data analysis, results interpretation and manuscript editing; BT and EP participated to data acquisition; MR and GB gave important contributions in data analysis, results interpretation, manuscript editing and final approval; CG was the medical physicist in charge of computing the dose and running the ExacTrac System; DA, GP were the physicians in charge of treatments; AP and RO gave final approval to conceptual study and manuscript.

All authors read and approved the final manuscript.

Authors declare that no competing interest exist

Authors declare that written informed consent was obtained from the patient for publication of this case report and accompanying images. A copy of the written consent is available for review by the Editor-in-Chief of this journal.
